# Dietary risk factors for colorectal cancer in Brazil: a case control study

**DOI:** 10.1186/s12937-016-0139-z

**Published:** 2016-02-27

**Authors:** Sandro Nunes Angelo, Gustavo J. Lourenço, Daniéla O. Magro, Helvia Nascimento, Rogério A. Oliveira, Raquel F. Leal, Maria de Lourdes S. Ayrizono, João J. Fagundes, Claudio S. R. Coy, Carmen S. P. Lima

**Affiliations:** 1 Department of Digestive Surgery, Faculty of Medical Sciences, Campinas State University, Campinas, São Paulo Brazil; 2 Department of Internal Medicine, Faculty of Medical Sciences, Campinas State University, Campinas, São Paulo Brazil; 3 Department of Preventive and Social Medicine, Faculty of Medical Sciences, Campinas State University, Campinas, São Paulo Brazil; 4 Department of Biostatistics, Bioscience Institute, Paulista State University, Botucatu, São Paulo Brazil

**Keywords:** Colorectal cancer, Dietary pattern, Lifestyle

## Abstract

**Background:**

High meat intake and low consumption of vegetables, fruits and whole grains have been associated with increased risk of colorectal cancer in some relevant cohort studies conducted in distinct ethnic populations. The role of the dietary pattern on the risk of sporadic colorectal adenocarcinoma (SCA) in Brazil is unknown; therefore, it was the aim of the present study.

**Methods:**

The dietary patterns of 169 patients with SCA and 101 controls were analysed by food frequency recall. Crude odds ratios were calculated and given within 95 % confidence intervals.

**Results:**

Patients reported higher average intakes of beef (32.0 ± 1.8 *versus* 23.7 ± 1.6, *P* = 0.0069), chicken (18.1 ± 0.9 *versus* 12.2 ± 0.8, *P* = 0.0002), and pork (8.9 ± 0.9 *versus* 3.4 ± 0.5, *P* < 0.0001). These individuals had a 1.025, 1.069, and 1.121-fold increased risk of SCA.

Similar consumption of fish, vegetables, fruits and whole grains was reported by patients and controls.

**Conclusions:**

Meat consumption is greater in patients with SCA in the Brazilian population. Considering the study population – characterized by ethnic heterogeneity –, the environmental factor related to food habits may be associated with higher incidence of this disease in Brazil.

## Background

Colorectal cancer (CRC) is result of a complex interaction of variables, including external factors, such as environmental and dietary agents, or internal ones, such as somatic or hereditary disorders [1]. Some environmental or lifestyle factors, including lack of exercise and smoking, seem to act directly as carcinogens or increase somatic mutation frequency, predisposing or determining the appearance of tumour [2].

There is a clear association between dietary pattern and CRC, but a specific carcinogen has not yet been identified to fully justify this relationship [3]. High red meat intake and low consumption of vegetables, fruits and whole grains have been associated with increased risk [4-11], and low red meat intake and high consumption of vegetables, fruits and whole grains [11-13] or high consumption of chicken and fish [4], with decreased risk of CRC, respectively, in some, but not in all [14, 15] relevant cohort studies conducted in distinct ethnic populations.

Cancer is the second cause of death in the Southeast region of Brazil [16] and CRC has a high incidence and mortality in the country [17]. Conversely, the ethnic origin of the Brazilian population is highly heterogeneous, consisting of indigenous Amerindian populations and immigrants from Europe, Africa and Asia [18, 19].

Therefore, it seemed interesting to evaluate the influence of dietary patterns on the risk of sporadic colorectal adenocarcinoma (SCA) in an ethnically heterogeneous population.

## Methods

### Patients and controls

This study comprised 169 patients with histologically confirmed SCA (97 males, 72 females; 140 Caucasians, 27 Blacks, 2 Asians; median age: 60 years, range: 29-87), diagnosed at the Research Ethics Committee, Faculty of Medical Sciences, Campinas State University. Patients with inflammatory bowel diseases, adenomatous polyposis, or a familial history of CRC were excluded from analysis. The control group comprised 101 blood donors (77 males, 24 females; 89 Caucasians, 12 Blacks; median age: 60 years, range: 55-67) from the same hospital, with no gastrointestinal symptoms, previous cancer or a familial history of CRC. The controls were randomly selected from the local population, according to the inclusion or exclusion criteria for age, gender and ethnic origin. All procedures were performed according to institutional guidelines, and patients and controls provided written informed consent. The local (document No. 227/2003) and the national (document No. 1544/2003) ethical committees approved the project.

### Dietary intake

Dietary habits of patients and controls were evaluated using a food-frequency questionnaire developed at the United States of America [20], which was previously validated to be used in Brazilian epidemiological studies [21]. The subjects were asked about the frequency of intake of various foods for the years before SCA onset or before the interview. The frequency of meat consumption was assessed separately for beef, chicken, pork, and fish ingestion.

**Fig. 1:**
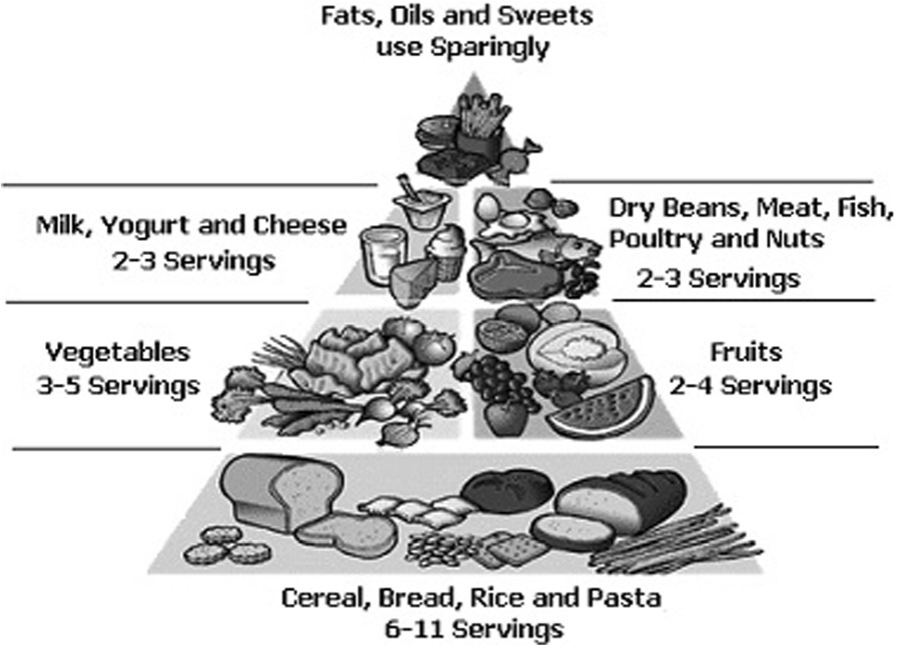
Brazilian food pyramid

The classification of patients with recommended, higher or less than recommended intake of meat, fruits, whole grains, vegetables and legumes was based on the Brazilian food pyramid (Fig. 1) criteria [22]. The portion considered for different types of meat in the analysis was 120 g.

### Statistical analysis

Statistical differences between groups were tested considering the significance level at 5 %. To assess the strength of the association between intakes of foods and risk of colorectal cancer, crude odds ratios (ORs) were calculated with 95 % confidence intervals. Patients and controls were compared according their dietary intakes in a case-control study. The multivariate logistic regression analysis was used to obtain adjusted ORs for different types of food. All analyses were performed using the SAS System 9.3 statistical package (SAS Institute Inc., Cary, NC, USA).

## Results

The demographic and lifestyle characteristics of SCA patients and controls enrolled in this study were similar and are presented in Table 1.

**Table 1:** Distribution of 169 patients with sporadic colorectal adenocarcinoma and 101 controls according to age, gender, ethic origin, smoking and alcoholism

	Patients (%)	Controls (%)	*P*-value
Age (years)			
<60	81 (47.9)	48 (47.5)	0.9487
≥60	88 (52.1)	53 (52.5)	
Gender			
Male	97 (57.4)	77 (76.2)	0.0018
Female	72 (42.6)	24 (23.8)	
Ethnic origin			
White	140 (82.8)	89 (88.1)	0.3435
Black	27 (16.0)	12 (11.9)	
Asian	02 (1.2)	0 (0.0)	
Smoking			
Never	78 (46.2)	57 (56.5)	0.1926
Past	47 (27.8)	26 (25.7)	
Current	44 (26.0)	18 (17.8)	
Alcoholism			
Never	128 (75.7)	83 (82.2)	0.3287
Past	21 (12.5)	7 (6.9)	
Current	20 (11.8)	11 (10.9)	

The average monthly intakes of the various food groups and ORs for SCA are presented in Table 2. Patients reported higher intakes of beef, chicken, and pork than controls. Similar intakes of fish, vegetables, fruits and whole grains were referred by patients and controls. An increased risk of SCA was associated with higher intakes of meat. Individuals with higher intakes of beef, chicken, and pork had a 1.025, 1.069, and 1.121-fold increased risk of disease when compared with controls.

**Table 2:** Distribution of 169 patients with sporadic colorectal adenocarcinoma and 101 controls according to intake of various food groups (logistic regression)

Food consumption	Patients mean^a^ ± SD	Controls mean^a^ ± SD	OR (95 % CI)	*P*-value
Beef	32.0 ± 1.8	23.7 ± 1.6	1.025 (1.007-1.044)	0.0069
Chicken	18.1 ± 0.9	12.2 ± 0.8	1.069 (1.032-1.108)	0.0002
Pork	8.9 ± 0.9	3.4 ± 0.5	1.121 (1.061-1.185)	<0.0001
Fish	5.1 ± 0.9	4.7 ± 0.6	0.999 (0.956-1.044)	0.9732
Vegetables	71.6 ± 3.3	76.8 ± 3.8	0.994 (0.986-1.001)	0.0995
Fruits	55.3 ± 3.5	63.7 ± 4.4	0.994 (0.988-1.001)	0.1137
Whole grains	1.2 ± 0.4	4.1 ± 1.0	0.963 (0.918-1.010)	0.1213

^a^mean number of portions per month

*SD* standard deviation, *OR* odds ratio, *CI* confidence interval

## Discussion

SCA is the third most frequent malignant neoplasm in western developed countries, but it has a lower incidence in undeveloped ones [23]. In Brazil, the estimated risk considered is 13 cases/100.000 for men and 15 cases/100.000 for women. It ranks the third most frequent internal cancer in men in the Southeast region of the country, whereas in the North it is the nineth [24].

The differences observed in the incidence among countries and regions suggest that environmental and diet aspects play important roles in carcinogenesis. To our knowledge, there are no studies in Brazil analysing dietary factors in SCA patients.

In this study, SCA patients had an expressive increase of meat intake, in their different types (beef, pork and chicken). This finding is similar to previous reports, which associated dietary aspects, such as high intake of red meat and saturated fat with low ingestion of fruits, vegetables, and grains containing soluble fiber and folic acid, and the increase in occurrence of SCA [9-11]. Within this context it is interesting to emphasize that studies in several countries [4-11, 25, 26] found a relation between this pattern of high consumption of meat and increased occurrence of SCA. Norat et al. performed a study of dietary patterns in ten European countries and found an increase in relative risk of SCA related to high consumption of meat and an inverse association with consumption of fish [6]. The association was also observed by Rodrigo & Riestra and Sanz et al. in Spain [7, 27], and by Turner et al. in the United Kingdom [5].

In Japan, Lee et al. related the ‘westernization’ of dietary habits in that country to high intakes of meat and low fiber, and the increased incidence of SCA [8]. Conversely, Kimura et al. found no relationship between the consumption of meat and the occurrence of SCA in the Japanese population [14]. These authors observed that the high consumption of fish may be related to a lower risk of the disease. An analysis of 14 prospective studies in the U.S. and Europe, by Koushik et al., did not confirm this association with high intake of meat [15].

Comparing the intake patterns of vegetables, whole grains and fruits in patient and control groups, there was no difference, emphasizing that they were lower than those recommended in the Brazilian food pyramid (Fig. 1) criteria [22] in both groups. Voorrips et al. and Michels et al. observed similar results without a relationship between reduced risk of SCA and vegetable and fruit consumption [28, 29]. Similar results were found by Rodrigo & Riestra in Spain, and by Schneider et al. in South Africa [7, 30]. Other studies [31, 32] showed that high intake of vegetables and fruits do not decrease the SCA risk; however, very low intake may be associated with SCA.

Key et al. evidenced in a review that the consumption of at least 400 g/day of vegetables and fruits was associated with a decreased risk of SCA in the American population [25]. Similar results were observed by Turner et al. and Yeh et al. [5, 12]. A review of over 700 works by Ryan-Harshman & Aldoori [13] and studies developed by Turner et al., Pou et al., and Yeh et al. [5, 11, 12] also showed that the consumption of fruits and vegetables works as a protection factor.

The findings of this study show that the intake of red meat, chicken and pork in patients with colorectal cancer is, respectively, 1.3, 1.5 and 2.6-times higher than in the control group. Considering the total meat consumption, the frequency of servings/day in patients with SCA and in the control group is, respectively, 2.54 and 1.74. Since the amount of protein per serving considered was 120 g, the average intake of animal protein in patients and controls was 304.8 g *vs*. 208.8 g, respectively. There was no difference related to fish, probably because the consumption of this food in the study population was relatively low.

Some limitations of the study were not considering other risk factors, such as obesity, and not obtaining information about body mass index.

In this study, the dietary patterns of SCA patients were similar to the population from the North hemisphere and Australia [4-7, 9, 25, 26]. This can be explained by the great influence of the North American and European countries on Brazilian food habits. The characterization of dietary habits and its correlation with SCA incidence in a Brazilian population may be important in the development of education and prevention programs.

## Conclusion

Meat consumption is greater in patients with SCA in the Brazilian population. Considering the study population – characterized by ethnic heterogeneity –, the environmental factor related to food habits may be associated with higher incidence of this disease in Brazil.

## Abbreviations

CRC: colorectal cancer
SCA: sporadic colorectal adenocarcinoma
